# Recent advances in microbial ε-poly-L-lysine fermentation and its diverse applications

**DOI:** 10.1186/s13068-022-02166-2

**Published:** 2022-06-16

**Authors:** Shubo Li, Yunren Mao, Lifei Zhang, Miao Wang, Jinhao Meng, Xiaoling Liu, Yunxia Bai, Yuan Guo

**Affiliations:** 1 College of Light Industry and Food Engineering, Guangxi University, Nanning, 530004 China; 2 National Engineering Research Center for Non-Food Biorefinery, Guangxi Academy of Sciences, Nanning, 530004 China

**Keywords:** ε-Poly-L-lysine, Metabolic regulatory mechanism, Antimicrobial mechanism, Functional properties, Fermentation performance

## Abstract

The naturally occurring homo-polyamide biopolymer, ε-poly-L-lysine (ε-PL) consists of 25–35 L-lysine residues with amide linkages between α-carboxyl groups and ε-amino groups. ɛ-PL exhibits several useful properties because of its unusual structure, such as biodegradability, water solubility, no human toxicity, and broad-spectrum antibacterial activities; it is widely applied in the fields of food, medicine, clinical chemistry and electronics. However, current industrial production of ε-PL is only performed in a few countries. Based on an analysis of the physiological characteristics of ε-PL fermentation, current advances that enhance ε-PL fermentation, from strain improvement to product isolation are systematically reviewed, focusing on: (1) elucidating the metabolic pathway and regulatory mechanism of ε-PL synthesis; (2) enhancing biosynthetic performance through mutagenesis, fermentation optimization and metabolic engineering; and (3) understanding and improving the biological activity and functional properties of ε-PL. Finally, perspectives on engineering and exploiting ε-PL as a source material for the production of various advanced materials are also discussed, providing scientific guidelines for researchers to further improve the ε-PL fermentation process.

## Introduction

As an important polycationic peptide, ε-Poly-L-lysine (ε-PL) is a homopolymer consisting of 25–35 L-lysine residues (molecular weight (Mw) range of 3,200–4,500 Da) with linkages between α-carboxyl and ε-amino groups [1, 2]. Because of the presence of many free amino groups in the main chain and many cationic amino groups on the side chains, ε-PL displays multication characteristics in acidic to slightly alkaline environments. In general, ε-PL is a slightly bitter tasting, hygroscopic, light-yellow natural cationic polymer, and can be completely digested into lysine by the body. Because of its novel structure, ɛ-PL exhibits several excellent physicochemical and biological properties, including water solubility, selective removal of endotoxins, anti-obesity properties, biodegradability, thermostability and nontoxicity toward humans and the environment [3–7]. More importantly, ε-PL exhibits broad-spectrum antimicrobial ability against Gram-negative and Gram-positive bacteria, such as Staphylococcus aureus, fungi, yeasts and some viruses [8], in which ε-PL strongly inhibits several food-borne pathogens, including *Escherichia coli* O157:H7 [9], *Listeria monocytogenes*, *Staphylococcus aureus*, *Serratia marcescens* [10]. Therefore, ε-PL and its derivatives have been of interest in preservatives, biodegradable fibers, hydrogels, drug carriers, biochip coatings in food and in the cosmetics and pharmaceutical fields [11, 12], making ɛ-PL has received considerable research and development attention [13, 14]. For example, based on its strong antibacterial activity, biodegradability and low toxicity, ε-PL has been generally recognized as a safe (GRAS No.000135) food preservative for routinely using in Japan, Korea, USA, China and other countries[15, 16].

Compared with chemical method, microorganisms are more practical, efficient and environment friendly for ɛ-PL production [17], and even microbial ε-PL exhibits more activity than chemically synthesized ε-PL [8]. As a secondary metabolite, ε-PL is mainly produced from sustainable resources such as sugars and glycerol through microbial fermentation by various *Streptomycetaceae*, a few filamentous fungi and some *Bacilli* [18, 19], but the production of ε-PL is unstable and dependent on cell density [20]. Recently, with the development of genetic engineering, bioinformatics, and advanced precision instruments and testing equipment, microbial ɛ-PL fermentation has been systematically investigated, focusing on screening of higher ɛ-PL-producing strains [21], development of new fermentation mode, regulation of the polymerization degree of ɛ-PL and understanding the biosynthetic mechanism of ɛ-PL [22], optimization of culture conditions [23], and illuminating the inhibitory effect of ε-PL on microorganisms [24]. Therefore, the intention of this review is to summarize recent developments in ɛ-PL fermentation, from strain-breeding to process optimization, focusing particularly on the update progress on the regulation mechanism of microbial ɛ-PL. In addition, perspectives on exploiting and modifying ε-PL to produce various advanced materials are also discussed, providing scientific guidelines and valuable insights for further enhancing the overall performance of ɛ-PL fermentation.

## Occurrence of ε-PL in microorganisms

### Assay methods for ε-PL

The most convenient and rapid method for determining ε-PL is a colorimetric procedure with the dye-methyl orange, which interacts with ε-PL to produce a water-insoluble compound, allowing the concentration of ε-PL to be calculated from the absorbance at 465 nm [25]. Recently, a series of qualitative, or quantitative methods have been developed to measure ε-PL concentration, including: (1) colorimetric methods with the acidic dye [26], e.g., molybdosilicate anion [27], poly R-478.24, or dipicrylamine [28]; (2) an improved high sensitivity colorimetric assay with glucose oxidase, in which ε-PL is determined without any pretreatment [29]; (3) integrating high-throughput screening strategy using ribosome engineering technology, permitting ε-PL to be determined in < 5 min per plate without compromising accuracy [30]; (4) high-performance liquid chromatography (HPLC) methods, which can determine the concentration and degree of polymerization (DP) of ε-PL. This wide choice of assays has permitted a large number of ε-PL-producing microorganisms to be screened, including various *Streptomycetes,* some *Bacilli* and a few filamentous fungi [31] (Table 1). Notably, *Streptomycetes* are considered as the best producers of ε-PL and commercial production of ε-PL is mainly depended on the fermentation with *Streptomyces albulus* [32, 33].

**Table 1:** ɛ-PL-producing strains and its fermentation performance

Strains(Origin)	Carbon and nitrogen sources	Fermentation strategy	Yield (g/L)	Productivity (g/L/h)	Detection method	Refs.
*Streptomyces* sp. M-Z18 (China)	Glycerol + fish meal + corn steep liquor	Addition of talc microparticles and acidic pH shock	62.36	0.329	Methyl orange precipitation method	[92]
*Streptomyces albulu*s FEEL-1 (China)	Glucose + yeast extract	Introducing related antibiotics	59.50	8.21 g/L/day	Methyl orange precipitation method	[145]
*Streptomyces albulus* W-156 (China)	Glycerol + yeast extract	Combining genome shuffling and gentamicin resistance	56.5	0.226	Methyl orange precipitation method	[76]
*Streptomyces* sp. AF3-44 (China)	Glycerol + yeast extract	Addition of exogenous glutathione	46.5	0.277	Methyl orange precipitation method	[104]
*Streptomyces* sp. FEEL-1 (China)	Glycerol + yeast extract	Screening by ARTP mutagenesis with streptomycin resistance	41.2	0.245	Methyl orange precipitation method	[75]
*Streptomyces* sp. M-Z18 (China)	Glycerol + beef extract	Screening ɛ-PL-tolerant strain by genome shuffling	39.96	0.231	Methyl orange precipitation method	[142]
*Streptomyces* sp. M-Z18 (China)	Industrial glycerol + beef extract	Through seed stage with in situ pH monitoring	36.22	0.195	Methyl orange precipitation method	[143]
*Streptomyces griseofuscus* (China)	Glucose + yeast extract	Addition of exogenous astaxanthin	36.1	0.188	Methyl orange precipitation method	[149]
*S. albulus* PD-1 (China)	Glucose + yeast extract	Overexpression of ammonium transporter gene	35.7	0.213	High-performance liquid chromatography	[146]
*S. albulus* PD-1 (China)	Glucose + yeast extract	Expression of VHb gene	34.2	0.204	High-performance liquid chromatography	[87]
*Kitasatospora* sp. MY 5–36 (China)	yeast extract + glucose	Immobilization on loofah sponge	34.11	9.34 g/L/day	High-perfor-mance liquid chromatography	[106]
*S. albulus* LS-84 (China)	Glucose + yeast extract + CSR	Intergeneric hybridization	32.6	0.166	Methyl orange precipitation method	[131]
*S. albulus* M-Z18 (China)	Glucose + yeast extract	Applying a novel two-stage fermentation	32.22	5.86 g/L/day	Methyl orange precipitation method	[82]
*Streptomyces ahygroscopicu*s GIM8 (China)	Glucose + yeast extract	One-Stage pH Control coupled with Nutrient Feeding	28.2	0.098	High-performance liquid chromatography	[97]
*Streptomyces.* sp. GIM8 (China)	Glucose + yeast extract	Resin-based in situ product removal strategy	23.4	0.117	Methyl orange precipitation method	[107]
*Streptomyces albulus* CICC 11,022 (China)	Glucose + yeast extract	OverexpressionofPLs and sodium citrate feeding	20.1	6.7 g/L/day	Mmethyl orange precipitation method	[101]
*S. albulus* S410 (Japan)	Glucose + yeast extract	A two stages pH control strategy	48.3	0.252	Methyl orange precipitation method	[77]
*S. albulus* NBRC14147 (Japan)	Polypeptone + yeast extract + sucrose	Inactivation of concomitant biosynthetic pathways	About 3.60	0.129	Methyl orange precipitation method	[14]
*Streptomyces noursei* NRRL 5126 (India)	proteose peptone, glycerol	Using the resting cell culture technique	2.36	0.028	Calorimetric determination by trypan blue precipitation	[144]
*Bacillus cereus* sp. (India)	Glucose + yeast extract	Adding metabolic precursors	0.565	0.006	Methyl orange precipitation method	[147]
*Bacillus licheniformis* L26 (India)	crude glycerol + peptone + yeast extract	Optimization of medium	155 mg/L	0.0013	Trypan blue precipitation method	[48, 148]

### Separation and purification of ε-PL

Few studies have focused on exploring methods for separating and purifying ε-PL from the fermentation medium [34, 35, 36]. In general, ε-PL exists in a fully protonated form (εPLH^n+^) in solution at pH < 4, but the εPLH^n+^ cation can interact with tetraphenylborate ion (TPB^–^) anion to form a precipitate, εPLH(TPB)n, at around pH 3.5. Addition of NaTPB precipitated both polycationic εPLH^n+^, and ammonium and potassium ions; the latter were removed from the mixture through washing with acetone. After dissolving εPLH(TPB)n in HCl solution, ε-PL was precipitated with acetone, as the hydrochloride salt, providing a rapid and simple method to purify basic peptides or polyamines [37]. Recently, an alternative strategy, including flocculation, filtration, ultrafiltration, ion-exchange chromatography, and decolorization, has been developed and applied to purify ε-PL from fermentation medium. Under the optimal conditions for adsorption and desorption on Amberlite IRC-50 resin, a final ε-PL with a purity of 92.4% was obtained with a 75% recovery [38]. Furthermore, an alcohol/salt aqueous two-phase system (ATPS) was also developed, in which an ATPS, consisting of 20% (w/w) ethanol and 20% (w/w) ammonium sulfate at pH 9.5, was applied to purify ε-PL from fermentation medium with triplicate extractions, and a final ε-PL product with 92.4% purity and 87.7% recovery was obtained with the combination of desalting by ultrafiltration [39].

### Metabolic pathway and regulation mechanism for microbial ε-PL

As a secondary metabolite, ε-PL was found in the culture filtrate of *S. albulus* No. 346 (currently designated *S. albulus* NBRC14147) [40], and mainly produced from sustainable resources such as sugars and glycerol by microbial fermentation by various *Streptomycetaceae*, such as *Streptomyces* MZ18 [18], *Streptomyces griseus* [19], *Streptomyces aureofaciens* [41], a few filamentous fungi and some *Bacilli* [18, 19] (Table 1).

#### Genome sequence for ɛ-PL-producing strains

In general, the synthesis of biopolymer is a sophisticated process that involves cell growth, precursor synthesis, energy provision, redox equilibrium, cofactor regulation, transportation of substrates and products, and complex global regulatory system [42]. Fortunately, the draft genome sequences of several ɛ-PL-producing strains (including *S. albulus* CCRC 11814 [43], *S. albulus* PD-1 [44], *S. albulus* NK660 [45], *Kitasatospora* sp. MY5-36 and *S. albulus* NBRC14147 [46] have been published. The genomes of the *Streptomycetes* exhibit high similarity, having genomes within the scope of 8.7 to 11.9 Mb and GC contents between 69 and 76% [42, 44]. However, the ɛ-PL-producing strains also have special characteristics; *S. albulus* ZPM has 44 gene clusters related to secondary metabolites, almost twice those of *Streptomyces coelicolor* A3 (25 gene clusters) [47]. In addition, *S. albulus* CCRC 11814 contains 69 tRNA genes and 4 rRNA genes with 1 rRNA operon located on contig 198, resulting in 9,177 protein-coding sequences being identified [48]. More importantly, *S. albulus* CCRC 11814 can produce enough L-lysine to support a high production level of ε-PL due to the lack of feedback regulation of aspartate kinase. Based on the genomic data, most genes encoding proteins for synthetic pathway, energy metabolic, transportation mechanism and regulation information in the ɛ-PL-producing strains have been successfully annotated, indicating that ɛ-PL is closely related to the biosynthesis and assembly of L-lysine, as well as being associated with multiple cellular processes [49]. Therefore, the genomic information was considered as a powerful platform for analyzing metabolic pathways and identifying candidate genes, enabling more complicated projects on metabolic analysis and pathway engineering, to further enhance the fermentation performance of ɛ-PL-producing strain.

#### Biosynthetic pathway for microbial ε-PL

**Fig. 1:**
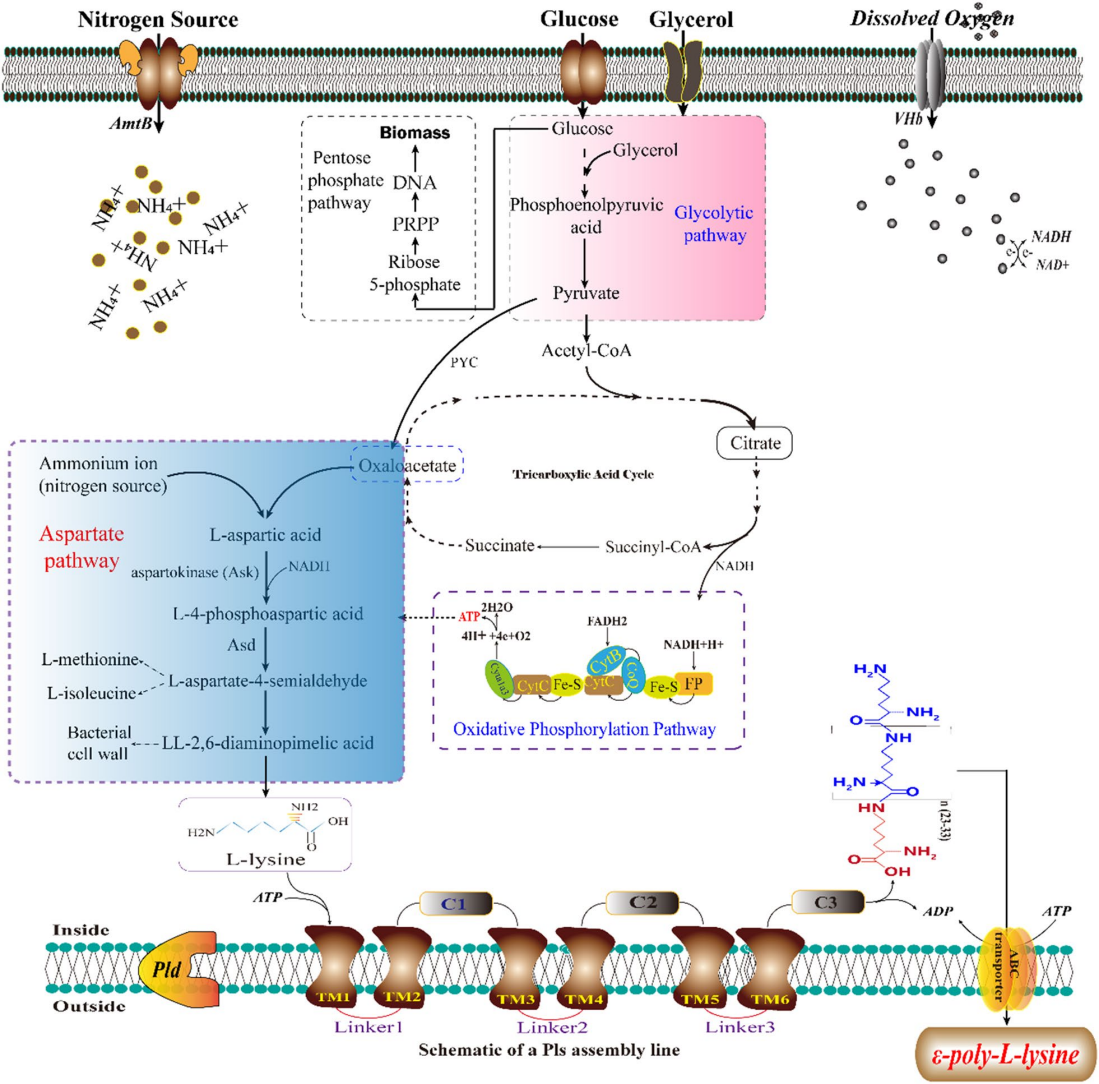
Microbial ε-PL biosynthetic pathway from glucose and glycerol [150, 151]. The domain architecture of ɛ-PL synthase (Pls), including six transmembrane domains (TM1 to TM6) and three tandem domains (C1–C3), is shown schematically [58]

As the direct monomeric precursor of ε-PL, L-lysine can be synthesized through the aspartate pathway from L-aspartate. As shown in Fig. 1, aspartokinase (Ask, catalyzing the phosphorylation of L-aspartic acid to form L-4-phosphoaspartic acid) and aspartate semialdehyde dehydrogenase (Asd, reducing L-4-phosphoaspartic acid into L-aspartate 4-semialdehyde) are the two key enzymes that regulate the synthesis of amino acids [50]. However, due to the complicacy of aspartate pathway, bacterial species have evolved diverse modes for Ask regulation, in which the Ask enzyme(s) of *S. albulus* appear to be resistant to feedback inhibition by L-lysine and/or L-threonine, thereby providing sufficient L-lysine for high-yield ε-PL biosynthesis [51].

In microorganisms, two mechanisms for the activation of amino acids are involved in peptide biosynthesis, i.e., adenylation by AMP, catalyzed by non-ribosomal peptide synthetases (NRPSs) and phosphorylation by ADP, catalyzed by amide ligases [52]. The mechanism of the assembly of L-lysine monomers into an ε-PL polymer is not completely understood, but it appears that ε-PL is biosynthesized in a manner similar to the action of AMP and NRPSs [53]. L-lysine monomers are adenylated on the carboxyl group, then transferred onto the ε-PL synthetase active site sulfhydryl group, to produce activated aminoacyl thioester intermediates [54]. The ε-PL synthetases (Pls) can catalyze repeated peptide bond formation reactions between the thio-esterified lysine and the ε-amino group of a free lysine, or of an ε-PL peptide, thereby progressively elongating the ε-PL [50, 55].

#### ε-PL synthetase (Pls) and ε-PL-degrading enzyme (Pld)

ε-PL synthetase is the essential enzyme for ε-PL synthesis (Fig. 1), but its catalytic mechanism was poorly understood until relatively recently. To probe the mechanism of Pls, some native enzymes were purified from the membrane fraction of *S. albulus*, and found that the affinity of L-lysine for Pls is relatively high. Pls is a single-module NRPS with the classic A- and T-domains of NRPSs, with six transmembrane (TM) domains surrounding three tandem soluble domains, without any thioesterase, or condensation domain, and acts as a ligase for peptide bond formation [56, 57]. During the biosynthesis of ε-PL, L-lysine monomers are polymerized via a three-step enzymatic reaction, involving adenylation, thiolation and peptide bond formation; and the catalytic mechanism is initiated at the N-terminus by the A- and T-domains with the adenylation and transfer of an incoming L-lysine monomer as an extending unit (described in detail by Hamano [58] and Kazuya [59]). The C-terminal tandem domains (C1-, C2- and C3-domains) catalyze peptide bond formation between the extending unit and a freely diffusible L-lysine molecule (priming unit) to produce an L-lysine dimer, then the dimer is used as a freely diffusible substrate (acceptor substrate) for the next polymerization reaction. The growing chain of ε-PL is never covalently bound to the enzyme, indicating that the enzyme holds the growing chain at the active site by non-covalent interactions, until the polymerization reactions are complete [58]. In summary, this catalytic cycle has no defined endpoint and Pls acts iteratively during ε-PL chain growth to produce a variety of chain lengths, normally between 25 and 35 residues. The linker regions of Pls are important in binding the growing chain of ε-PL to the active site and providing insights into their function, i.e., connecting the TM domains and regulating the chain length of ε-PL [56]. Although a detailed description of the process that the growing polymer interacts with the enzyme remains unclear, the improved understanding of the physiological function of Pls has contributed to the creation of new classes of biopolymers, by biosynthetic engineering.

Furthermore, Pls exhibited a typical hyperbolic saturation curve with respect to the L-lysine concentration at a constant ATP concentration during the catalytic reaction, indicating that Pls is allosterically regulated by ATP [60]. However, the affinity of Pls for ATP is lower than that of L-lysine (approximately 2 mM) and at low concentrations (0.25–2 mM) and high concentrations (3–5 mM) of ATP, the enzyme exhibits negative and positive allosteric interactions, respectively, indicating that Pls requires a high concentration (> 3 mM) of ATP for full activity [60]. However, no Pls polymerization catalytic activity was tested with other nucleotides, such as GTP, CTP, and TTP. Therefore, ATP, generated by the glycolytic pathway and electron-transport chain, is accumulated as an important cofactor for ε-PL biosynthesis, in which allosteric regulation of Pls avoids excessive consumption of ATP for primary metabolism.

Since ε-PL has antibiotic activity, ε-PL producers generally produce a membrane-bound ε-PL-degrading enzyme (Pld), which degrades ɛ-PL to protect the producing organism. During the ε-PL fermentation, ε-PL can be accumulated when the pH decreases to around 3.2, but Pld activity is detectable at neutral pH; Pld acts via an exopeptidase-type mode, releasing N-terminal L-lysine residues one by one [60]. However, although *S. virginiae* IFO12827 and *S. norsei* IFO15452 have high ε-PL degrading activity, both strains can still produce a high yield of ε-PL, indicating an inverse correlation between the distributions of membrane-bound Pls and Pld [61]. More importantly, a high level of ε-PL-degrading activity was also found in a *pld* (encoding ε-PL-degrading enzyme) knockout mutant, suggesting that ε-PL degradation into shorter chains is also catalyzed by endo-peptidases, without the release of L-lysine monomers [60]. Recently, the gene encoding an unidentified ε-PL-degrading enzyme (Pld II), with endo-type peptidase activity, was identified in *S. albulus* NBRC14147 [60], and the Pld II activity was found on the cell surface, indicating that Pld II is bound to the outside of the cytoplasmic membrane. However, a knockout mutant of both pldI and pldII still could not produce ε-PL under neutral pH conditions [60]. Therefore, an acidic pH is an important factor, both to activate Pls and inhibit ε-PL degradation by Pld, as well as accumulating intracellular ATP, resulting in the production and accumulation of ε-PL [60, 56].

#### Regulation of the peptide chain length of microbial ɛ-PL

Similar to other biopolymers, the biological functions of ε-PL closely rely on its molecular weight, so the ability to produce it with a specific chain length, or a defined range of chain lengths, would be very beneficial to widen the potential applications of ε-PL [62]. Currently, the commercial ε-PL commonly ranges from 3.0 to 4.5 kDa (corresponding to 25–35-mer) [48] and this chain-length diversity is generated directly by the synthetase, rather than the degrading enzyme [56, 63]. However, despite the improved understanding of the mechanism of ε-PL biosynthesis, the processes that control the polymerization degree of ε-PL are still not fully elucidated, resulting in few commercially available ε-PLs with controlled molecular weight ranges. At present, five strategies have been applied to selectively produce short-chain ε-PL, but all have drawbacks: (1) Screening strains for ability to produce short chain (5–20 residues) ε-PL, but this approach gives low yields (4.5 g/L) [64, 65]; (2) Adding aliphatic short chain polyols, or cyclodextrins (such as sulfated cyclodextrin) to the growth medium, to regulate the chain-length [66] or molecular weight [67] of ε-PL, but such polyols present food safety risks; (3) Shortening the chain length by engineering ε-PL-synthetase through mutagenesis of its linker regions [58], but the yield is low and the synthesis mechanism is not fully understood; (4) Using ε-PL-degrading enzymes to produce short chain ε-PL from naturally occurring ε-PL [68], but consistent control of the chain length between different batches is difficult, the enzymes are costly and the process is inefficient; (5) Modification of fermentation conditions, using glycerol as carbon source, can produce short chain ε-PL (8–32 residues) in high yield (39.8 g/L), high purity (98.8%) and high recovery ratio (72.6%); however, the chain length range is wide and poorly controlled [69].

## Strategies for improving the performance of ε-PL fermentation

During the conventional ε-PL fermentation, the viscosity of culture medium increases with the accumulation of ε-PL, requiring slow and difficult processing to separate the ε-PL from the bacterial cells, thereby decreasing the process yield. Furthermore, lysis of the bacterial cells during fermentation makes semi-continuous culture difficult and significantly increases the production costs of ε-PL, restricting industrial production to a few countries. To meet the rapidly increasing demand for ɛ-PL, various approaches have been employed to enhance the synthetic efficiency of ε-PL, achieving higher yields in less time.

### Breeding higher yielding ε-PL-producing strains by mutagenesis

In the aspartate pathway, L-lysine can partially repress the synthesis of aspartokinase and glycine inhibits aspartokinase activity. Therefore, some resistant mutants (S-(2-aminoethyl)-L-cysteine and glycine-resistant) were screened for reduced feedback inhibition, increased cell growth and increased ε-PL production. For example, resistant mutant was screened using sulfaguanidine, glycine, L-lysine and DL-3 hydroxynorvaline as selection markers, in which *S. diastatochromogenes* L9 was selected and produced 0.77 g/L ε-PL, 15% higher than that of the original strain [70]. Meanwhile, genome shuffling has also been applied to simultaneously improve the glucose tolerance, ε-PL tolerance and ε-PL productivity of *S. graminearus* [71, 72]. With the help of genome shuffling in five species (*Streptomyces padanus*, *Streptomyces griseofuscus*, *Streptomyces graminearus*, *Streptomyces hygroscopicus* and *Streptomyces albulus*), the hybrid FEEL-1 was identified through morphology and spore color, and ε-PL production correspondingly increased to 1.12 g/L, about 2.75-fold higher than that of the control [73]. Furthermore, mutagenesis combined with streptomycin resistance (such as streptomycin, gentamicin, and rifampicin) was also developed to improve the fermentation performance of ε-PL-producing strains [74, 75]. For example, after atmospheric and room temperature plasma (ARTP) mutagenesis and genome shuffling, *S. albulus* AG3-28 was screened with an increased concentration of gentamicin and could produce 3.43 g/L of ε-PL with a lower molecular weight in shake-flask culture [76].

### Optimization of fermentation processes to improve ε-PL production

The original ɛ-PL fermentation strategy was the two-phase pH control approach, in which the culture medium pH is maintained above 5.0 for optimal cell growth, then lowered to pH 4.0 for optimal ɛ-PL biosynthesis, giving an ɛ-PL yield of 48.3 g/L [77]. However, because of the toxicity of ε-PL to its own producer organism and the largely unknown self-protection mechanism, further enhancing ɛ-PL production is very difficult. Several fermentation strategies have been developed to increase ɛ-PL production, including: (1) The pH control strategy, (2) the dissolved oxygen control strategy, (3) optimization of the fermentation medium and (4) improvement of the fermentation conditions. These strategies have significantly improved ɛ-PL production and decreased the overall processing costs [31] and are discussed in detail below.

#### Improving ɛ-PL production by pH-control

In the conventional ε-PL fermentation, ε-PL could be only accumulated when the pH was below 5.0 and the maximum synthesis rate was detected at about pH 4.0 [78]. Therefore, strict pH control strategy is essential to achieve high ε-PL production, in which low pH increases membrane permeability and prevents the binding of ε-PL onto the cells [79]. More importantly, when the ε-PL-producing strain was subjected to acid stress, the enhancement of acid tolerance was contributed to improved fermentation performance [80]. According to the analysis of specific cell growth and the rate of ε-PL production, a novel two-stage pH control method was developed by changing the culture pH from 3.5 to 3.8 after 36 h, which increased the ε-PL yield and productivity from 7.8 g/L and 3.1 g/L/day to 9.1 g/L and 4.8 g/L/day, respectively [81]. Meanwhile, after evaluation of the effects of acidic pH-shock on mycelial metabolic activity, an integrated pH-shock strategy was proposed, involving a pre-acid shock adaptation period at pH 5.0, followed by an acidic pH 3.0 shock for 12 h (including a gradual pH decrease from 4.0 to 3.0), finally increasing the pH to 4.0 to promote ε-PL accumulation. As a result, both cell growth and ɛ-PL yield were greatly improved, increasing the maximum ε-PL yield and productivity to 54.70 g/L and 6.84 g/L/day, respectively [78]. Similarly, another novel two-stage fermentation, including culture and fermentation stages, was also developed based on the analysis of conventional pH shock fermentation, achieving ε-PL yield and productivity increases to 32.22 g/L and 5.86 g/L/day, which were 32.3% and 36.6% higher, respectively, than those of the conventional fermentation [82].

In summary, the fermentation performance of ε-PL can be remarkably improved by pH shock strategy, but the underlying mechanism is poorly understood [83]. Based on the analysis of transcriptomic and physiological data, several physiological mechanisms have been proposed (described in detail by Wang [80]), as follows [84, 85]: (1) Changes in cellular morphology during fed-batch fermentation, providing a predictor for determining the ε-PL production rate; (2) physiological changes (such as the enhancement of mycelial respiration and up-regulation of key genes involved in central metabolic and ε-PL biosynthetic pathways) induced by acidic pH shock have contributed synergistically to the improvement of ε-PL biosynthesis [84]; (3) the synthesis of ε-PL is a cellular response to acid stress, providing new insights into enhancing ε-PL production via adaptive evolution or metabolic engineering [80]; (4) signal transduction systems play a major role in responding to pH shock, enhancing metabolic vitality and intracellular redox homeostasis [85]. (5) pH shock influences a wide range of proteins including global regulators, fatty acid desaturase, respiratory chain components and ATP-binding cassette transporter, thereby improving cellular respiratory activity and enhancing ε-PL productivity [83].

#### Improving the production of ɛ-PL by dissolved oxygen control strategy

The dissolved oxygen (DO) level is another key factor for ɛ-PL fermentation, in which the high DO level is beneficial for the formation of ATP, and then improves cell growth and ɛ-PL biosynthesis. A DO control strategy, with a fixed DO level at 40% during the cell growth phase, followed by a reduction to 20% in the ɛ-PL biosynthesis phase, increased the biomass and ɛ-PL production to 1.99 and 20.73 g/L, respectively [23]. However, due to the intertwined hyphae, high cell density and high ε-PL yield, the culture medium becomes viscous, resulting in decreased oxygen transfer efficiency [86], making it hard to increase the DO level by simply enhancing agitation and aeration. In addition, stronger agitation and higher shear stress could result in undesired effects on mycelial morphology, ε-PL productivity and the polymerization degree of ε-PL. Therefore, addition of oxygen-vectors may be an effective strategy to improve the oxygen supply during the ε-PL fermentation, in which 0.5% n-dodecane maintained DO levels above 32% saturation, and then increased the ε-PL production from 23.4 to 30.8 g/L [49]. Similarly, with the introduction of *Vitreoscilla hemoglobin* gene in the chromosome of *S. albulus* PD-1, the capability of binding oxygen was correspondingly enhanced for *S. albulus* PD-1, making the ε-PL production increased by 50.7% than that of wild-type strain [87].

#### Improving the production of ɛ-PL by fermentation medium optimization

In ε-PL fermentation, the most common culture medium is Medium 3G (M3G), which contains a high proportion of glucose, with the most expensive component being yeast extract [88]. To decrease the cost of ε-PL fermentation, alternative carbon sources (such as glycerol, or molasses) were used to produce ɛ-PL [89, 90]. A mixed glucose/glycerol carbon source accelerated cell growth and L-lysine biosynthesis and greatly shortened the fermentation time, resulting in a higher yield of ε-PL and biomass [91]. For example, glycerol as carbon source effectively enhanced ɛ-PL production, reaching 62.36 g/L in a 5-L bioreactor after 192 h [92]. However, the positive effects of mixed carbon sources on ε-PL production were still incompletely understood [93]. Consequently, chemostat culture was applied to investigate the ability of ε-PL biosynthesis with diverse carbon sources at the same dilution rate. Glucose and glycerol were consumed synergistically to accelerate energy metabolism and carbon fluxes, resulting in a higher energy charge and NADH/NAD^+^ ratio, providing sufficient carbon skeletons and ATP to supply precursors and energy for high ε-PL production [94, 95]. However, the simultaneous consumption of glucose and glycerol generated a high level of reactive oxygen species (ROS) in the batch fermentation stage, damaging cells and decreasing the specific ε-PL formation rate, suggesting that intensive cellular respiration enhanced ε-PL production [96].

As a result of the self-inhibition of cell growth by ε-PL, insufficient biomass can become another key problem during the ε-PL fermentation. To deal with this problem, several nitrogen-rich nutrients were added after 16 h of culture, of which the addition of 0.5% (w/v) of yeast extract increased ε-PL production to 2.24 g/L. Meanwhile, the final ε-PL yield was further increased to 28.2 g/L by coupling a two-stage pH control strategy with continuous supplementation of yeast extract at 0.5 g/h [97]. However, organic nitrogen (yeast extract) is the most expensive component of the medium for ɛ-PL production, but selection of alternative nitrogen sources to replace yeast extract has contributed to decreasing the cost of ɛ-PL fermentation. Therefore, agro-industrial by-products, i.e., fish meal and corn steep liquor, have been applied as alternative nitrogen sources for industrial ɛ-PL fermentation. With the combination of optimized medium and two-stage pH control, a cost-effective and efficient process for ɛ-PL production was developed in the fed-fermentation, in which the yield and productivity of ɛ-PL increased to 35.24 g/L and 4.85 g/L/day, respectively [86].

#### Improving ε-PL production by the addition of exogenous substances

As shown in Fig. 1, L-lysine is the direct precursor for ε-PL biosynthesis, and addition of 3 mM L-lysine or D-lysine could effectively improve the fermentation performance, increased the ε-PL production to 1.16 g/L and 1.20 g/L, respectively, which were 41.4% and 46.3% increments over control [98]. More importantly, with the combination of adding L-lysine with glucose–glycerol co-fermentation, the production of ε-PL could be further increased to 37.6 g/L under optimal conditions, suggesting that the bottleneck of ε-PL synthesis is the availability of L-lysine [99]. Furthermore, high ATP levels are essential for optimal Pls activity, and the tricarboxylic acid cycle is the main source of ATP [60]. Therefore, addition of some intermediates (such as L-aspartic acid and citric acid) can not only promote the accumulation of intracellular L-lysine, but also provides a substrate to produce sufficient ATP [100]. For example, the addition of citric acid inhibited the metabolic pathway from phosphoenolpyruvate to the TCA cycle, and consequently enhanced the metabolic flux from phosphoenolpyruvate to oxaloacetate and L-aspartate, thereby improving ε-PL production [100]. However, even though the levels of L-lysine and ATP are high enough, ε-PL cannot be accumulated efficiently if there is insufficient Pls activity. With the synergistic effect of 2 g/L sodium citrate, the ε-PL yield of the pls gene overexpressing strain (Q-PL2) were 211% higher than that of the wild strain. During the fed-batch fermentation, recombinant Q-PL2 produced 20.1 g/L of ε-PL in 72 h, which was 3.2-fold than that of wild-type strain. Therefore, ε-PL synthase is one of the rate-limiting enzymes in the ε-PL synthesis pathway [101].

Iron, manganese and cobalt can be added to the growth medium to regulate ε-PL biosynthesis [102, 103]. For example, adding 10 g/L of talc microparticles improved mycelial morphology and ε-PL production by decreasing pellet diameter from 297.63 to 205.65 μm and increasing ε-PL yield from 1.67 g/L to 2.51 g/L. Combination of talc microparticles with acidic pH shock increased the yield of ε-PL to 62.36 g/L from 54.70 g/L after 192 h [92]. However, the toxicity of ε-PL, significantly decreased the growth-vigor of *Streptomyces* as ε-PL accumulated and sharply increased the level of reactive oxygen species (ROS) after 24 h. Addition of antioxidants (such as glutathione and astaxanthin) further enhanced ε-PL production by limiting the increase in ROS [104]. Addition of 1.0 g/L astaxanthin increased ε-PL production by 36.3% to 36.1 g/L, suggesting that adding an antioxidant maintains cell vigor by reducing the excess ROS, and thus, improving the ε-PL yield [105].

To minimize the self-inhibition of ε-PL on cell growth, the immobilization approach has been developed and applied to ε-PL fermentation by entrapment or adsorption of *Kitasatospora* sp. on bagasse, synthetic sponge, macro-porous silica gel, or loofah sponge. As a result, the immobilized cells on loofah sponge could be reused five times over a period of 526 h, and lag phase was only detected in the first batch and the fermentation period was significantly shortened, increasing the yield and productivity of ε-PL from 22.53 g/L and 3.30 g/L/day to 34.11 g/L and 9.34 g/L/day, respectively [106]. More importantly, combining cell immobilization with in situ adsorption (ISA) of ɛ-PL was able to overcome the feedback inhibition and toxic effects of the accumulated ɛ-PL, in which loofah sponge-immobilized cells combined with ISA produced 3.64 g/L of ε-PL compared with 2.73 g/L by free cells combined with ISA [107, 108].

### Improvement of ε-PL production by metabolic engineering

In the post-genomic era, genetic engineering has become an important method to improve the fermentation performance of industrial microbial strains. However, up to now, few researches employing genetic engineering have been carried out to improve ε-PL production, as follow: (1) enhancing the efficiency of oxygen transfer during the ε-PL fermentation. To alleviate oxygen limitation, the *Vitreoscilla* hemoglobin gene (*vgb*) was integrated into the chromosome of *S. albulus* PD-1, increasing the ε-PL yield from 22.7 to 34.2 g/L with a productivity of 4.9 g/L/day [87]. Similarly, with the overexpression of heterologous *vgb* and SAM synthetase gene (*metK*) in *S. albulus* NK660, strain *S. albulus* NK660-VHb could produce 26.67% higher ε-PL and 14.57% higher biomass than the wild-type strain, respectively [109]; (2) improving the efficiency of nitrogen translocation and utilization. With the overexpression of ammonium transporter gene *amt*B, the production of ε-PL could be significantly increased from 22.7 g/L to 35.7 g/L when the optimum carbon-to-nitrogen ratio increased from 3 to 4.71 in the synthesis stage of fermentation [110]; (3) engineering ε-PL synthase to break the rate-limiting polymerization reaction [111, 112], in which ε-PL synthase was over-expressed in *S. albulus*, and then the recombinant Q-PL2 could produce 88.2% more than that of wild strain [101]. However, only two ɛ-PL-producing strains (*S. albulus* IFO14147 and *S. albulus* PD-1) have well-developed gene delivery systems, seriously limiting the potential for genetic modification of ɛ-PL-producing strains [60]. Therefore, a deeper understanding of the regulation, interactions with other metabolic pathways and mechanism of ɛ-PL biosynthesis, could facilitate a rational genetic approach, integrating high-throughput techniques, omics data and in silico models, and then provide extensive information on the regulatory and biosynthetic systems of ε-PL production and promote enhanced metabolic flux towards ε-PL biosynthesis [14].

## Antimicrobial mechanism of ε-PL and its functional modification

**Fig. 2:**
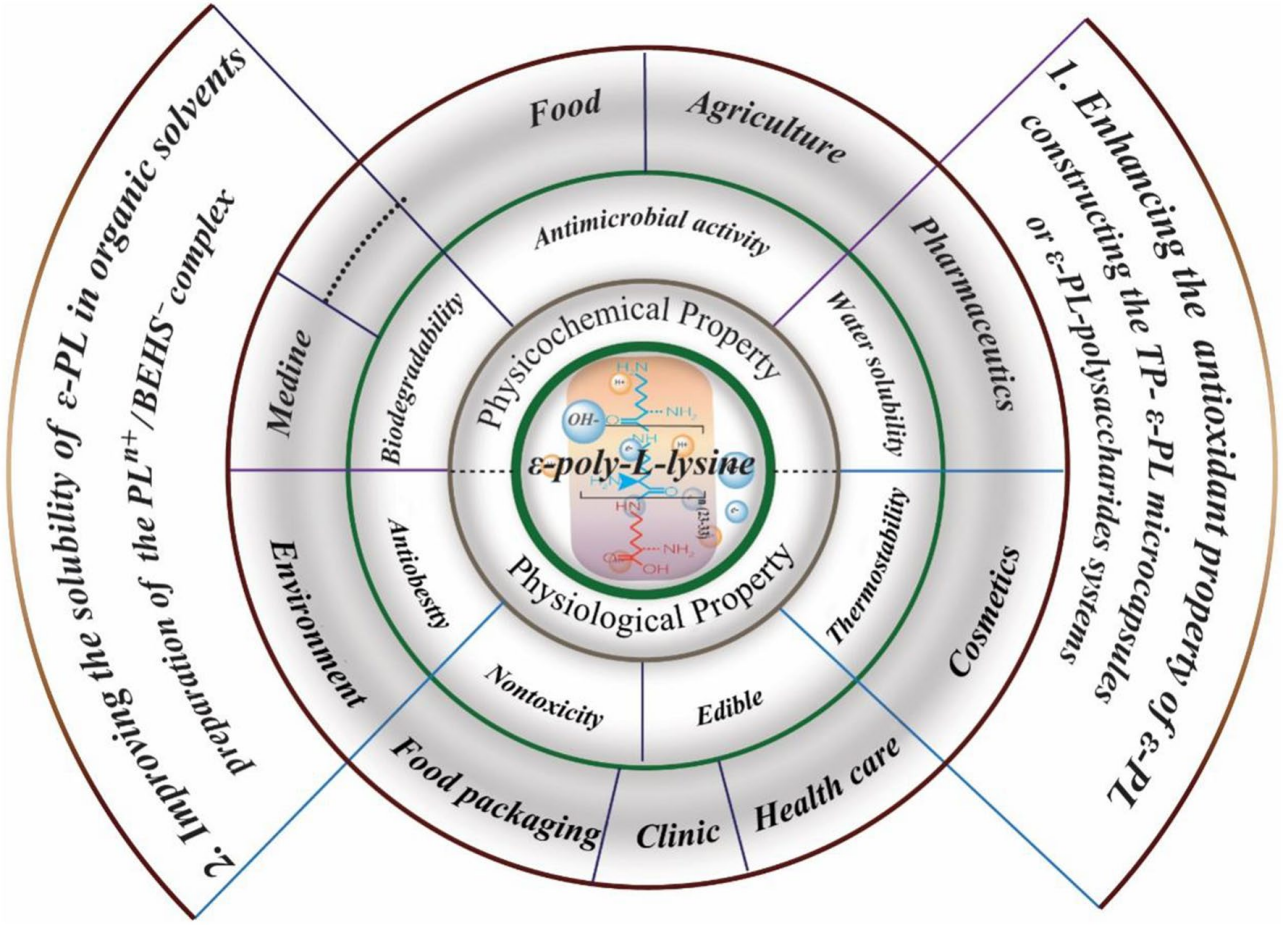
Various applications of ε-PL and modification strategies for broadening ε-PL application potential [117]

Compared with synthetic chemical preservatives, ɛ-PL exhibits biodegradability, water solubility and no human toxicity, and has been approved as a commercial food preservative by the U.S. Food and Drug Administration and equivalent organizations in several other countries [33, 113, 114]. ε-PL has been successfully used in the fields of food preservation (reviewed in detail by Tuersuntuoheti et al. [13], anti-microbial treatments [115, 116, 117], food packaging [118], control of environmental pathogens [119], medicine (reviewed in detail by Shukla et al. [120]) and agriculture (Fig. 2). However, the antimicrobial activity of ɛ-PL is closely related to its molecular weight (MW), and the antimicrobial mechanisms of different MW ranges are different and not well-understood. In general, low-MW ε-PL (< 1 kDa) slightly damages the cell membrane/wall and weakly inhibits the glycolytic pathway, but high-MW ε-PL (1–3 kDa and > 3 kDa) causes cell-wall lesions and increases cell membrane permeability, resulting in protoplasm leakage and cell death [121]. More importantly, the antimicrobial activity of ε-PL can be markedly reduced by chemical modification of its α-amino groups, the presence of anionic polymers, or alkaline conditions [116]. However, the antimicrobial activity of ε-PL can be enhanced by the presence of some water-soluble and biocompatible materials, such as chitosan oligomers or food additives [122]. In summary, ε-PL has a complex antimicrobial mode of action, with multiple cellular targets and mechanisms, which are not well-understood.

**Fig. 3:**
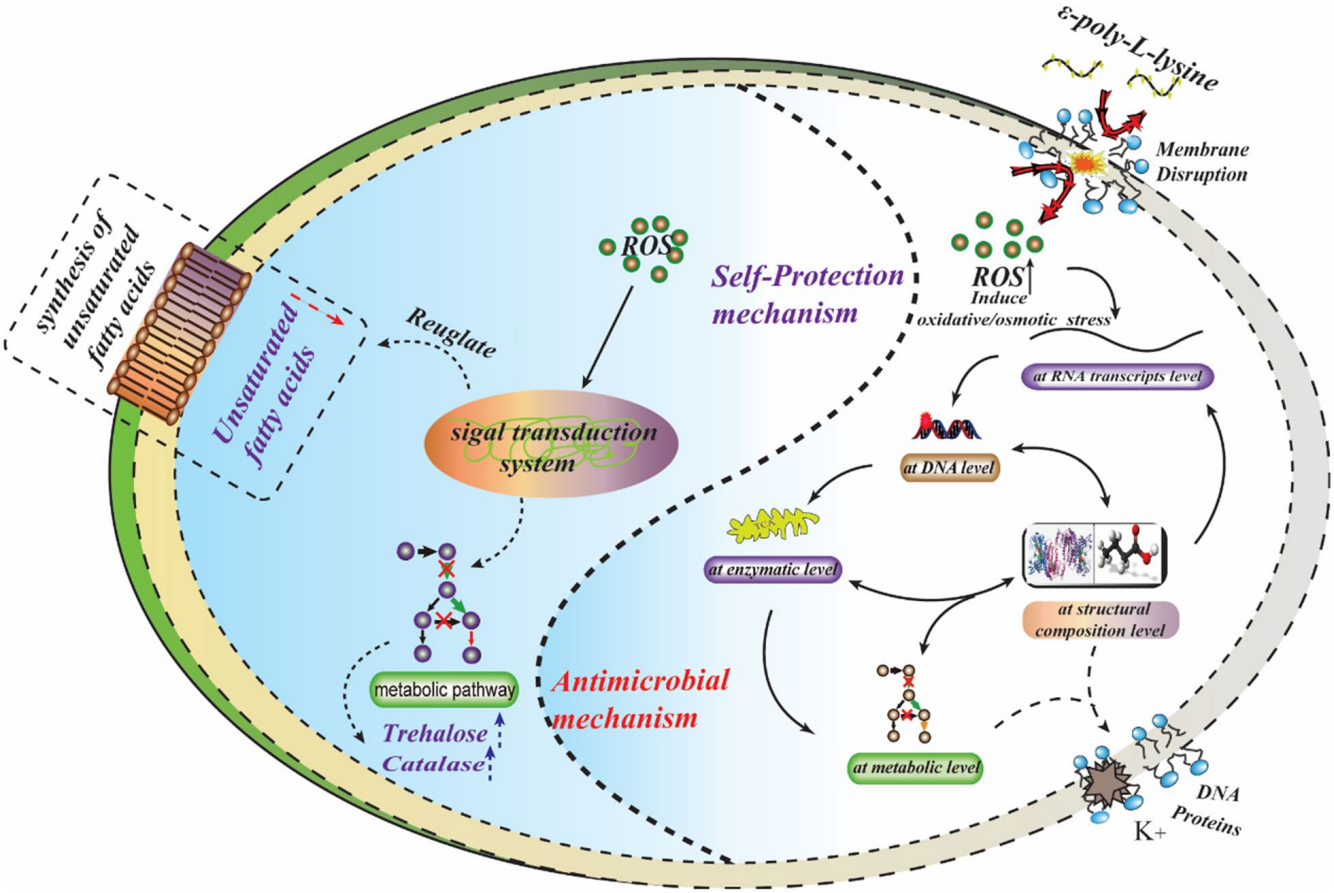
Potential antibacterial mechanisms of ε-PL and the self-protection mechanism of ε-PL-producing organisms. Extracellular ε-PL can disrupt the cell membrane, increasing its permeability and causing leakage of cytoplasmic material. Intracellular ε-PL increases the level of reactive oxygen species (ROS), resulting in damage to genomic DNA and enzymes. Both extra- and intra-cellular mechanisms can result in cell death [152, 126, 140]

At present, membrane disruption is the main antimicrobial mode of action of ε-PL and appears to operate by a number of potential mechanisms, i.e.: (1) electrostatic adsorption onto the cell surface [123]. The cationic nature of ε-PL allows it to adsorb onto the cell membrane, disrupting membrane integrity, decreasing respiratory activity, and damaging cell walls and membranes [124, 125]; (2) changing the membrane fatty acid and cell wall composition, resulting in leakage of electrolytes and proteins [116]; (3) inducing osmotic stress by disrupting the ionic balance between the inside and outside of the membrane [116]; and (4) stimulating the accumulation of intracellular reactive oxygen species (ROS), decreasing the integrity of the cell wall and membrane [126, 127]. In summary, cell membrane disruption and generation of oxidative stress, are the two general modes of action involved in ε-PL antimicrobial activity (Fig. 3).

There are, however, some limitations to the application of ε-PL. Because of the polycationic nature, ε-PL has poor antioxidant properties, which confers strong binding affinity to anionic materials and affects its antibacterial activity. Modification with several monosaccharides, using the Maillard reaction increased its DPPH radical scavenging capacity by 40.5–69.4%, increased its reducing power (from 0.9 to 1.3) with low cytotoxicity, and caused a slight decrease in antimicrobial activity [128]. Tea polyphenols, micro-encapsulated with ε-PL, had long-acting and slow-release antibacterial properties, with potential applications in food preservation [129].

The antimicrobial activity of ε-PL is modulated by interaction with various biopolymers with different charge distributions, both when there is little binding interaction, e.g., with polycationic chitosan or neutral dextran, and when there are strong electrostatic interactions and/or complex formation, e.g., with anionic carrageenan, alginate, or pectin [130, 131]. For example, ε-PL can interact with polysaccharide and/or protein components in food [132], but the polysaccharides protect ε-PL’s antimicrobial activity [129, 133]. Reacting a mixture of ɛ-PL and D-fructose produced ɛ-PL/fructose complexes with antimicrobial activity, low toxicity and biodegradability, as well as enhanced mechanical properties and water resistance [134].

ɛ-PL forms useful antimicrobial polycationic chains (PL^n+^) in water because of its high water-solubility. This high water-solubility limits the applicability of ɛ-PL in biopolymer-based plastics, but preparation of a complex (PL^n+^/BEHS^−^) between ɛ-PL and an anionic surfactant, bis(2-ethylhexyl) sulfosuccinate (BEHS^−^) made the ɛ-PL soluble in organic solvents, permitting its use as a coating material to produce a water-resistant antimicrobial membrane. In addition, the thermoplastic PL^n+^/BEHS^−^ complex was able to be uniformly mixed with polypropylene by heating, creating several new materials with antimicrobial activities [135]. Similarly, a series of dialdehyde microcrystalline cellulose (DAMC) particles, crosslinked with ɛ-PL (ɛ-PL–DAMCs) was prepared by reacting DAMC with varying amounts of ɛ-PL. The ɛ-PL–DAMC complexes had broad-spectrum antibacterial activity, suggesting great potential for use in food packaging [118].

## Conclusions

As an important biopolymer, ɛ-PL has several excellent properties (such as water solubility, no human toxicity, biodegradability and broad-spectrum antibacterial activity), and has been widely used in the chemical, food, environmental, medical, biotechnology and other industries. Since the discovery of ɛ-PL in 1977, great progress in fermentation improvement has been made through selective-breeding of producing strains, combined with process optimization [11, 136, 137]. The rapidly increasing demand for ɛ-PL has attracted increasing research interest in ε-PL fermentation, resulting in the development of a number of bioprocesses for effectively producing ε-PL [137, 138]. However, ɛ-PL synthesis is a complex process, involving cell growth, precursor biosynthesis, energy metabolic, redox equilibria, and transportation of substrates and products, making the metabolic pathway of ɛ-PL is strictly regulated by complicated global regulatory systems. More importantly, little is known about the catalytic mechanisms of ɛ-PL synthase (Pls) and ε-PL-degrading enzyme (Pld) [58, 139], how the polymerization of L-lysine to ɛ-PL is regulated at the molecular level, or how regulatory interactions control the chain length of ε-PL [63, 140]. Therefore, there are considerable knowledge gaps relating to various aspects of ε-PL production and downstream processing, resulting in current industrial production of ε-PL being carried out in only a few countries, and very few ε-PL products with controlled molecular weight ranges available commercially.

Considering the huge potential of ε-PL in various applications, there is considerable interest in further improving the economic viability of ε-PL production by fermentation. Future research should follow a three-pronged approach, involving upstream (strain improvement), midstream (advanced fermentation strategies) and downstream (cost-effective recovery) processes (Tuersuntuoheti et al., 2019) [141]. Specific research targets include:

- Considering the intrinsic complexity of ε-PL biosynthesis, targeting global regulatory mechanisms and transcription factors to further enhance microbial production performance (i.e., superior pH tolerance, higher ε-PL yield and production rate, and precise molecular weight control) at the global level by integrating systems biology and synthetic biology.
- Creating a superior ε-PL bioprocess by integrating fermentation and downstream processes, such as the use of immobilization supports and continuous-flow systems, the design of suitable bioreactors, and coupling with *in situ* methods for extraction and recovery, achieving a higher yield, in less time, at reduced cost.
- Modify the structure and composition of ε-PL by biotechnological approaches, to improve its physicochemical properties and broaden its application potential.

## Data Availability

Not applicable.
